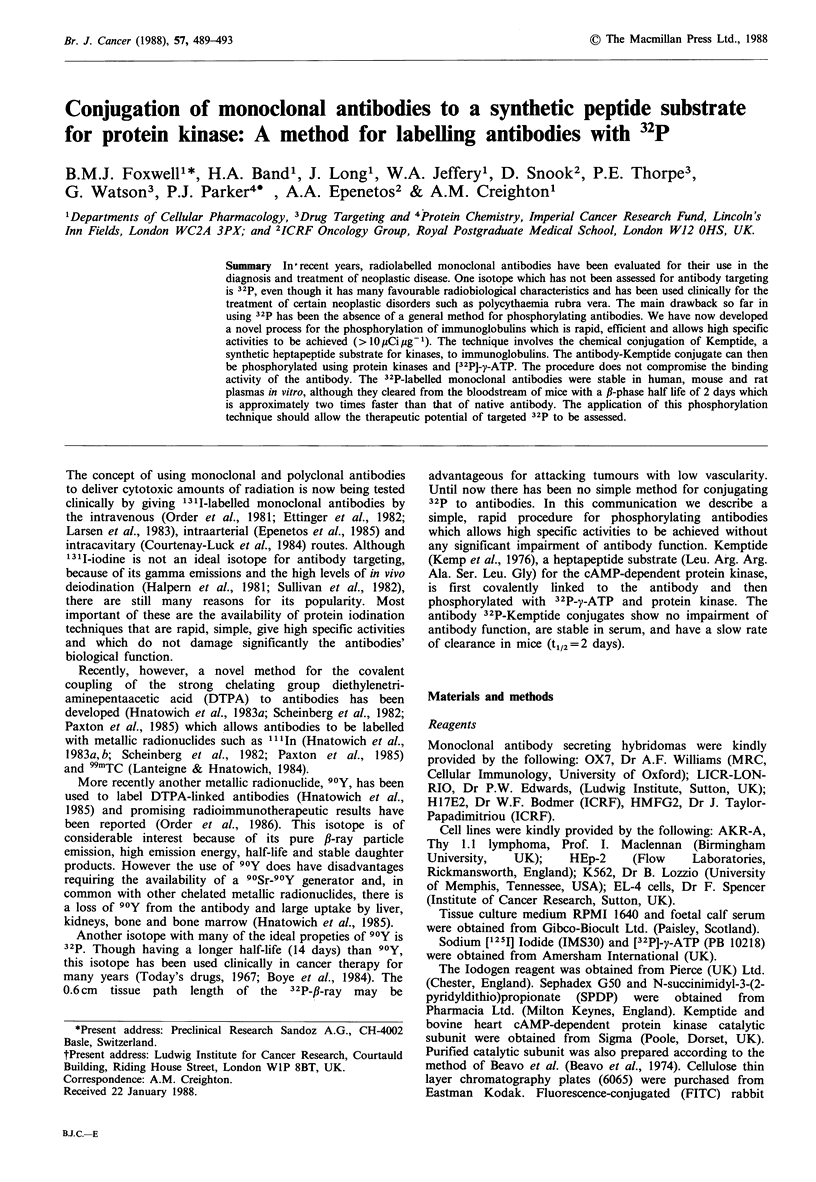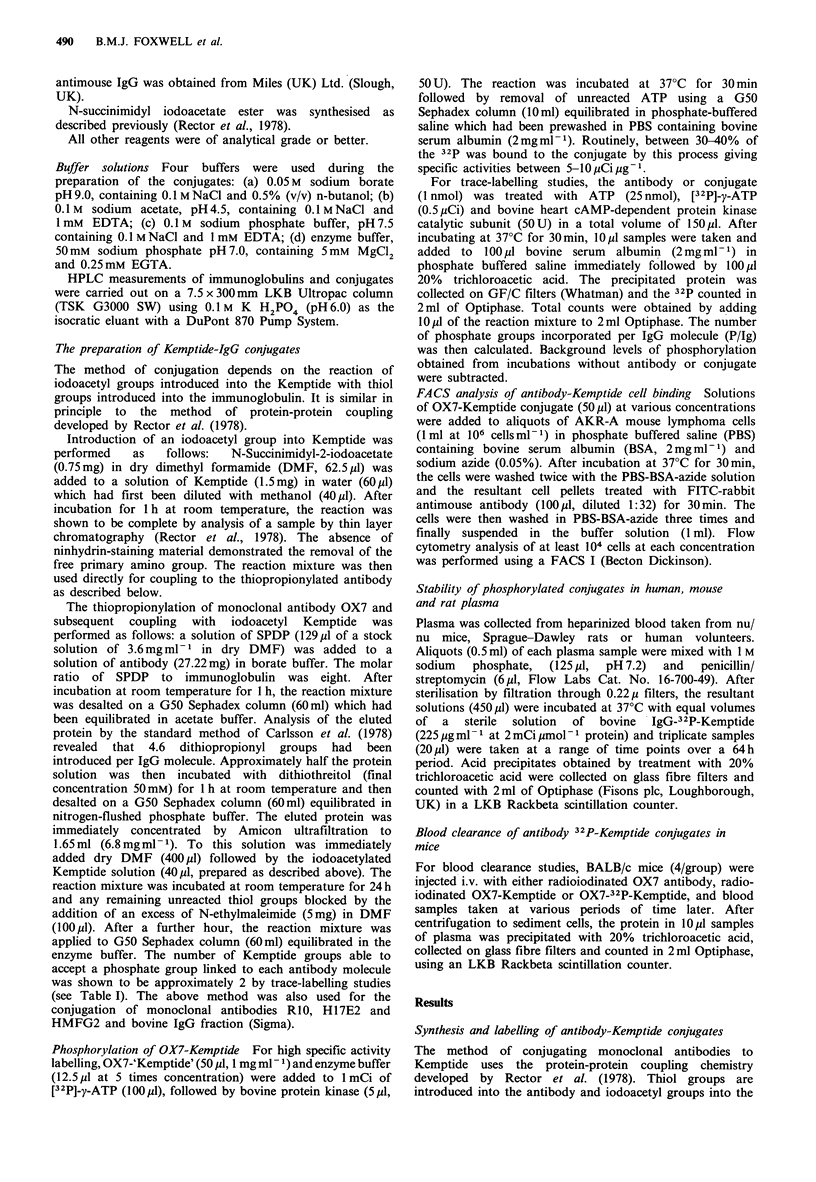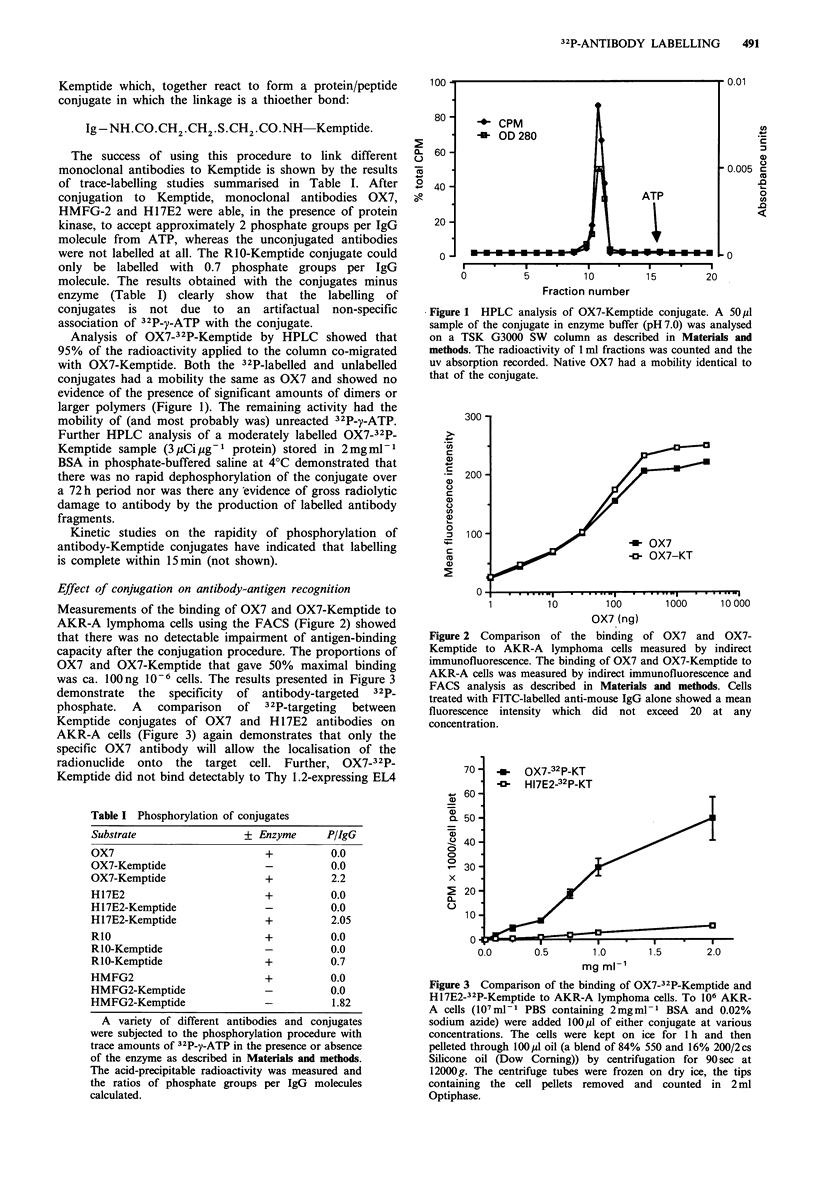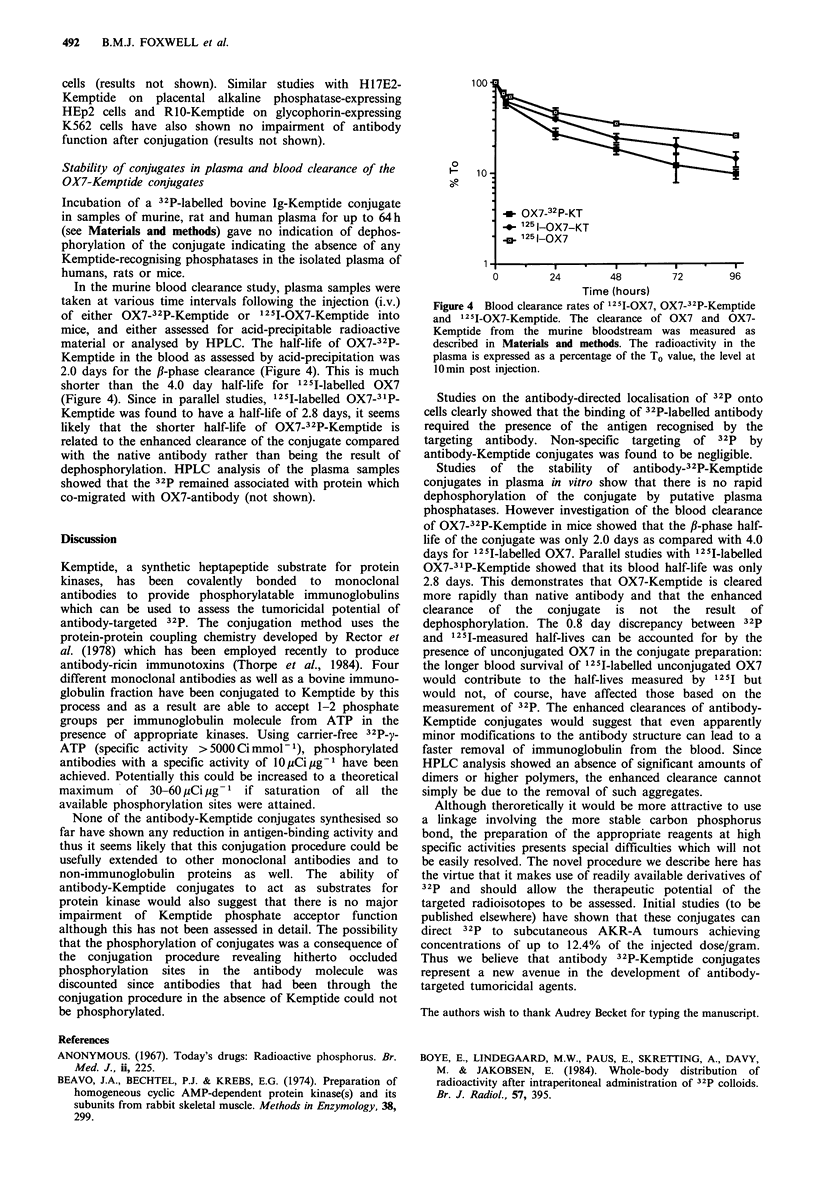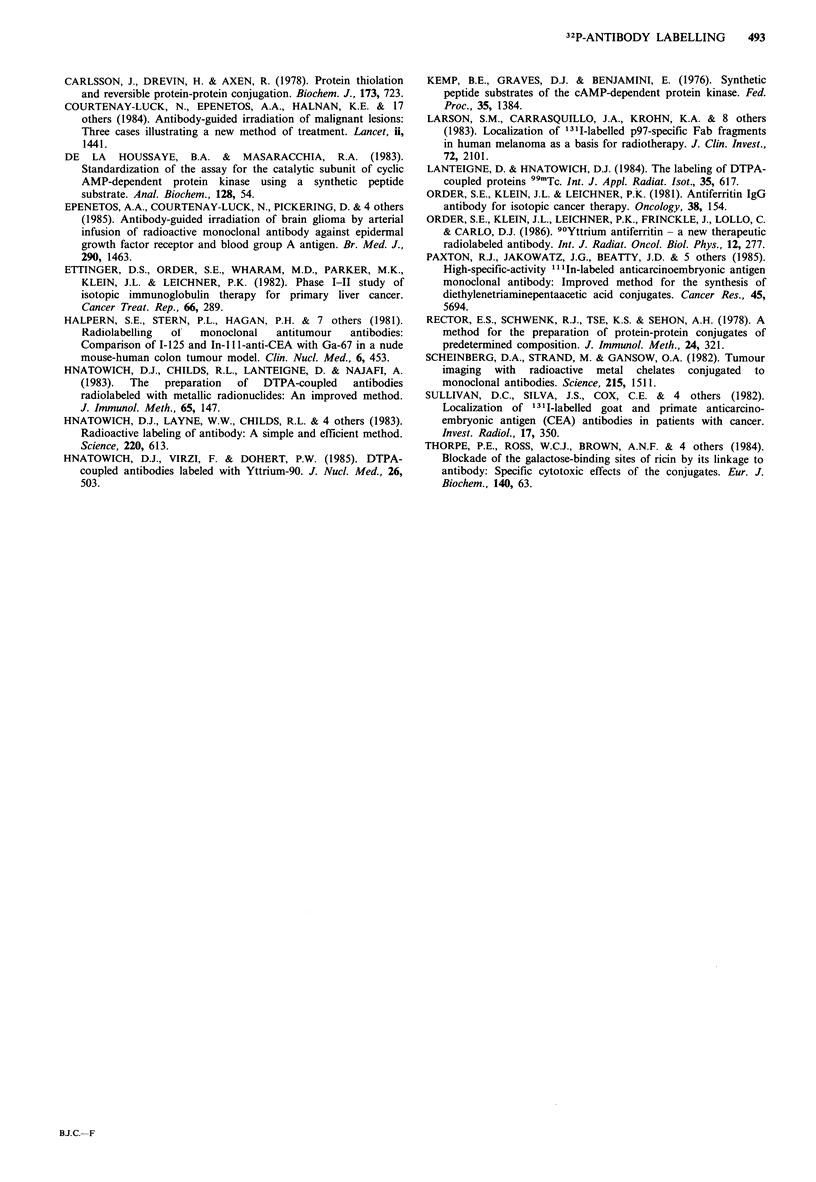# Conjugation of monoclonal antibodies to a synthetic peptide substrate for protein kinase: a method for labelling antibodies with 32P.

**DOI:** 10.1038/bjc.1988.112

**Published:** 1988-05

**Authors:** B. M. Foxwell, H. A. Band, J. Long, W. A. Jeffery, D. Snook, P. E. Thorpe, G. Watson, P. J. Parker, A. A. Epenetos, A. M. Creighton

**Affiliations:** Department of Cellular Pharmacology, Imperial Cancer Research Fund, London, UK.

## Abstract

In recent years, radiolabelled monoclonal antibodies have been evaluated for their use in the diagnosis and treatment of neoplastic disease. One isotope which has not been assessed for antibody targeting is 32P, even though it has many favourable radiobiological characteristics and has been used clinically for the treatment of certain neoplastic disorders such as polycythaemia rubra vera. The main drawback so far in using 32P has been the absence of a general method for phosphorylating antibodies. We have now developed a novel process for the phosphorylation of immunoglobulins which is rapid, efficient and allows high specific activities to be achieved (greater than 10 muCi micrograms-1). The technique involves the chemical conjugation of Kemptide, a synthetic heptapeptide substrate for kinases, to immunoglobulins. The antibody-Kemptide conjugate can then be phosphorylated using protein kinases and [32P]-gamma-ATP. The procedure does not compromise the binding activity of the antibody. The 32P-labelled monoclonal antibodies were stable in human, mouse and rat plasmas in vitro, although they cleared from the bloodstream of mice with a beta-phase half life of 2 days which is approximately two times faster than that of native antibody. The application of this phosphorylation technique should allow the therapeutic potential of targeted 32P to be assessed.


*Br. J. Cancer* (1988), **57**, 489–493 © The Macmillan Press Ltd., 1988

### Conjugation of monoclonal antibodies to a synthetic peptide substrate for protein kinase: A method for labelling antibodies with $^{32}P$

B.M.J. Foxwell[1]*, H.A. Band[1], J. Long[1], W.A. Jeffery[1], D. Snook[2], P.E. Thorpe[3], G. Watson[3], P.J. Parker[4]• , A.A. Epenetos[2] & A.M. Creighton[1]

[1]*Departments of Cellular Pharmacology,* [3]*Drug Targeting and* [4]*Protein Chemistry, Imperial Cancer Research Fund, Lincoln's Inn Fields, London WC2A 3PX; and* [2]*ICRF Oncology Group, Royal Postgraduate Medical School, London W12 0HS, UK.*

**Summary** In recent years, radiolabelled monoclonal antibodies have been evaluated for their use in the diagnosis and treatment of neoplastic disease. One isotope which has not been assessed for antibody targeting is $^{32}P$, even though it has many favourable radiobiological characteristics and has been used clinically for the treatment of certain neoplastic disorders such as polycythaemia rubra vera. The main drawback so far in using $^{32}P$ has been the absence of a general method for phosphorylating antibodies. We have now developed a novel process for the phosphorylation of immunoglobulins which is rapid, efficient and allows high specific activities to be achieved ($>10\,\mu Ci\,\mu g^{-1}$). The technique involves the chemical conjugation of Kemptide, a synthetic heptapeptide substrate for kinases, to immunoglobulins. The antibody-Kemptide conjugate can then be phosphorylated using protein kinases and [$^{32}P$]-$\gamma$-ATP. The procedure does not compromise the binding activity of the antibody. The $^{32}P$-labelled monoclonal antibodies were stable in human, mouse and rat plasmas *in vitro*, although they cleared from the bloodstream of mice with a $\beta$-phase half life of 2 days which is approximately two times faster than that of native antibody. The application of this phosphorylation technique should allow the therapeutic potential of targeted $^{32}P$ to be assessed.

The concept of using monoclonal and polyclonal antibodies to deliver cytotoxic amounts of radiation is now being tested clinically by giving $^{131}I$-labelled monoclonal antibodies by the intravenous (Order *et al.*, 1981; Ettinger *et al.*, 1982; Larsen *et al.*, 1983), intraarterial (Epenetos *et al.*, 1985) and intracavitary (Courtenay-Luck *et al.*, 1984) routes. Although $^{131}I$-iodine is not an ideal isotope for antibody targeting, because of its gamma emissions and the high levels of *in vivo* deiodination (Halpern *et al.*, 1981; Sullivan *et al.*, 1982), there are still many reasons for its popularity. Most important of these are the availability of protein iodination techniques that are rapid, simple, give high specific activities and which do not damage significantly the antibodies' biological function.

Recently, however, a novel method for the covalent coupling of the strong chelating group diethylenetriaminepentaacetic acid (DTPA) to antibodies has been developed (Hnatowich *et al.*, 1983*a*; Scheinberg *et al.*, 1982; Paxton *et al.*, 1985) which allows antibodies to be labelled with metallic radionuclides such as $^{111}In$ (Hnatowich *et al.*, 1983*a,b*; Scheinberg *et al.*, 1982; Paxton *et al.*, 1985) and $^{99m}TC$ (Lanteigne & Hnatowich, 1984).

More recently another metallic radionuclide, $^{90}Y$, has been used to label DTPA-linked antibodies (Hnatowich *et al.*, 1985) and promising radioimmunotherapeutic results have been reported (Order *et al.*, 1986). This isotope is of considerable interest because of its pure $\beta$-ray particle emission, high emission energy, half-life and stable daughter products. However the use of $^{90}Y$ does have disadvantages requiring the availability of a $^{90}Sr$-$^{90}Y$ generator and, in common with other chelated metallic radionuclides, there is a loss of $^{90}Y$ from the antibody and large uptake by liver, kidneys, bone and bone marrow (Hnatowich *et al.*, 1985).

Another isotope with many of the ideal propeties of $^{90}Y$ is $^{32}P$. Though having a longer half-life (14 days) than $^{90}Y$, this isotope has been used clinically in cancer therapy for many years (Today's drugs, 1967; Boye *et al.*, 1984). The 0.6 cm tissue path length of the $^{32}P$-$\beta$-ray may be advantageous for attacking tumours with low vascularity. Until now there has been no simple method for conjugating $^{32}P$ to antibodies. In this communication we describe a simple, rapid procedure for phosphorylating antibodies which allows high specific activities to be achieved without any significant impairment of antibody function. Kemptide (Kemp *et al.*, 1976), a heptapeptide substrate (Leu. Arg. Arg. Ala. Ser. Leu. Gly) for the cAMP-dependent protein kinase, is first covalently linked to the antibody and then phosphorylated with $^{32}P$-$\gamma$-ATP and protein kinase. The antibody $^{32}P$-Kemptide conjugates show no impairment of antibody function, are stable in serum, and have a slow rate of clearance in mice ($t_{1/2}=2$ days).

### Materials and methods

### *Reagents*

Monoclonal antibody secreting hybridomas were kindly provided by the following: OX7, Dr A.F. Williams (MRC, Cellular Immunology, University of Oxford); LICR-LON-RIO, Dr P.W. Edwards, (Ludwig Institute, Sutton, UK); H17E2, Dr W.F. Bodmer (ICRF), HMFG2, Dr J. Taylor-Papadimitriou (ICRF).

Cell lines were kindly provided by the following: AKR-A, Thy 1.1 lymphoma, Prof. I. Maclennan (Birmingham University, UK); HEp-2 (Flow Laboratories, Rickmansworth, England); K562, Dr B. Lozzio (University of Memphis, Tennessee, USA); EL-4 cells, Dr F. Spencer (Institute of Cancer Research, Sutton, UK).

Tissue culture medium RPMI 1640 and foetal calf serum were obtained from Gibco-Biocult Ltd. (Paisley, Scotland).

Sodium [$^{125}I$] Iodide (IMS30) and [$^{32}P$]-$\gamma$-ATP (PB 10218) were obtained from Amersham International (UK).

The Iodogen reagent was obtained from Pierce (UK) Ltd. (Chester, England). Sephadex G50 and N-succinimidyl-3-(2-pyridyldithio)propionate (SPDP) were obtained from Pharmacia Ltd. (Milton Keynes, England). Kemptide and bovine heart cAMP-dependent protein kinase catalytic subunit were obtained from Sigma (Poole, Dorset, UK). Purified catalytic subunit was also prepared according to the method of Beavo *et al.* (Beavo *et al.*, 1974). Cellulose thin layer chromatography plates (6065) were purchased from Eastman Kodak. Fluorescence-conjugated (FITC) rabbit

*Present address: Preclinical Research Sandoz A.G., CH-4002 Basle, Switzerland.
†Present address: Ludwig Institute for Cancer Research, Courtauld Building, Riding House Street, London W1P 8BT, UK.
Correspondence: A.M. Creighton.
Received 22 January 1988.

antimouse IgG was obtained from Miles (UK) Ltd. (Slough, UK).

N-succinimidyl iodoacetate ester was synthesised as described previously (Rector *et al.*, 1978).

All other reagents were of analytical grade or better.

*Buffer solutions* Four buffers were used during the preparation of the conjugates: (a) 0.05 M sodium borate pH 9.0, containing 0.1 M NaCl and 0.5% (v/v) n-butanol; (b) 0.1 M sodium acetate, pH 4.5, containing 0.1 M NaCl and 1 mM EDTA; (c) 0.1 M sodium phosphate buffer, pH 7.5 containing 0.1 M NaCl and 1 mM EDTA; (d) enzyme buffer, 50 mM sodium phosphate pH 7.0, containing 5 mM $MgCl_2$ and 0.25 mM EGTA.

HPLC measurements of immunoglobulins and conjugates were carried out on a 7.5 x 300 mm LKB Ultropac column (TSK G3000 SW) using 0.1 M K $H_2PO_4$ (pH 6.0) as the isocratic eluant with a DuPont 870 Pump System.

*The preparation of Kemptide-IgG conjugates*

The method of conjugation depends on the reaction of iodoacetyl groups introduced into the Kemptide with thiol groups introduced into the immunoglobulin. It is similar in principle to the method of protein-protein coupling developed by Rector *et al.* (1978).

Introduction of an iodoacetyl group into Kemptide was performed as follows: N-Succinimidyl-2-iodoacetate (0.75 mg) in dry dimethyl formamide (DMF, 62.5 µl) was added to a solution of Kemptide (1.5 mg) in water (60 µl) which had first been diluted with methanol (40 µl). After incubation for 1 h at room temperature, the reaction was shown to be complete by analysis of a sample by thin layer chromatography (Rector *et al.*, 1978). The absence of ninhydrin-staining material demonstrated the removal of the free primary amino group. The reaction mixture was then used directly for coupling to the thiopropionylated antibody as described below.

The thiopropionylation of monoclonal antibody OX7 and subsequent coupling with iodoacetyl Kemptide was performed as follows: a solution of SPDP (129 µl of a stock solution of 3.6 mg $ml^{-1}$ in dry DMF) was added to a solution of antibody (27.22 mg) in borate buffer. The molar ratio of SPDP to immunoglobulin was eight. After incubation at room temperature for 1 h, the reaction mixture was desalted on a G50 Sephadex column (60 ml) which had been equilibrated in acetate buffer. Analysis of the eluted protein by the standard method of Carlsson *et al.* (1978) revealed that 4.6 dithiopropionyl groups had been introduced per IgG molecule. Approximately half the protein solution was then incubated with dithiothreitol (final concentration 50 mM) for 1 h at room temperature and then desalted on a G50 Sephadex column (60 ml) equilibrated in nitrogen-flushed phosphate buffer. The eluted protein was immediately concentrated by Amicon ultrafiltration to 1.65 ml (6.8 mg $ml^{-1}$). To this solution was immediately added dry DMF (400 µl) followed by the iodoacetylated Kemptide solution (40 µl, prepared as described above). The reaction mixture was incubated at room temperature for 24 h and any remaining unreacted thiol groups blocked by the addition of an excess of N-ethylmaleimide (5 mg) in DMF (100 µl). After a further hour, the reaction mixture was applied to G50 Sephadex column (60 ml) equilibrated in the enzyme buffer. The number of Kemptide groups able to accept a phosphate group linked to each antibody molecule was shown to be approximately 2 by trace-labelling studies (see Table I). The above method was also used for the conjugation of monoclonal antibodies R10, H17E2 and HMFG2 and bovine IgG fraction (Sigma).

*Phosphorylation of OX7-Kemptide* For high specific activity labelling, OX7-'Kemptide' (50 µl, 1 mg $ml^{-1}$) and enzyme buffer (12.5 µl at 5 times concentration) were added to 1 mCi of [$^{32}$P]-γ-ATP (100 µl), followed by bovine protein kinase (5 µl, 50 U). The reaction was incubated at 37°C for 30 min followed by removal of unreacted ATP using a G50 Sephadex column (10 ml) equilibrated in phosphate-buffered saline which had been prewashed in PBS containing bovine serum albumin (2 mg $ml^{-1}$). Routinely, between 30–40% of the $^{32}$P was bound to the conjugate by this process giving specific activities between 5–10 µCi $\mu g^{-1}$.

For trace-labelling studies, the antibody or conjugate (1 nmol) was treated with ATP (25 nmol), [$^{32}$P]-γ-ATP (0.5 µCi) and bovine heart cAMP-dependent protein kinase catalytic subunit (50 U) in a total volume of 150 µl. After incubating at 37°C for 30 min, 10 µl samples were taken and added to 100 µl bovine serum albumin (2 mg $ml^{-1}$) in phosphate buffered saline immediately followed by 100 µl 20% trichloroacetic acid. The precipitated protein was collected on GF/C filters (Whatman) and the $^{32}$P counted in 2 ml of Optiphase. Total counts were obtained by adding 10 µl of the reaction mixture to 2 ml Optiphase. The number of phosphate groups incorporated per IgG molecule (P/Ig) was then calculated. Background levels of phosphorylation obtained from incubations without antibody or conjugate were subtracted.

*FACS analysis of antibody-Kemptide cell binding* Solutions of OX7-Kemptide conjugate (50 µl) at various concentrations were added to aliquots of AKR-A mouse lymphoma cells (1 ml at $10^6$ cells $ml^{-1}$) in phosphate buffered saline (PBS) containing bovine serum albumin (BSA, 2 mg $ml^{-1}$) and sodium azide (0.05%). After incubation at 37°C for 30 min, the cells were washed twice with the PBS-BSA-azide solution and the resultant cell pellets treated with FITC-rabbit antimouse antibody (100 µl, diluted 1:32) for 30 min. The cells were then washed in PBS-BSA-azide three times and finally suspended in the buffer solution (1 ml). Flow cytometry analysis of at least $10^4$ cells at each concentration was performed using a FACS I (Becton Dickinson).

*Stability of phosphorylated conjugates in human, mouse and rat plasma*

Plasma was collected from heparinized blood taken from nu/nu mice, Sprague–Dawley rats or human volunteers. Aliquots (0.5 ml) of each plasma sample were mixed with 1 M sodium phosphate, (125 µl, pH 7.2) and penicillin/streptomycin (6 µl, Flow Labs Cat. No. 16-700-49). After sterilisation by filtration through 0.22 µ filters, the resultant solutions (450 µl) were incubated at 37°C with equal volumes of a sterile solution of bovine IgG-$^{32}$P-Kemptide (225 µg $ml^{-1}$ at 2 mCi $\mu mol^{-1}$ protein) and triplicate samples (20 µl) were taken at a range of time points over a 64 h period. Acid precipitates obtained by treatment with 20% trichloroacetic acid were collected on glass fibre filters and counted with 2 ml of Optiphase (Fisons plc, Loughborough, UK) in a LKB Rackbeta scintillation counter.

*Blood clearance of antibody $^{32}$P-Kemptide conjugates in mice*

For blood clearance studies, BALB/c mice (4/group) were injected i.v. with either radioiodinated OX7 antibody, radioiodinated OX7-Kemptide or OX7-$^{32}$P-Kemptide, and blood samples taken at various periods of time later. After centrifugation to sediment cells, the protein in 10 µl samples of plasma was precipitated with 20% trichloroacetic acid, collected on glass fibre filters and counted in 2 ml Optiphase, using an LKB Rackbeta scintillation counter.

### Results

*Synthesis and labelling of antibody-Kemptide conjugates*

The method of conjugating monoclonal antibodies to Kemptide uses the protein-protein coupling chemistry developed by Rector *et al.* (1978). Thiol groups are introduced into the antibody and iodoacetyl groups into the

Kemptide which, together react to form a protein/peptide conjugate in which the linkage is a thioether bond:

$$Ig-NH.CO.CH_2.CH_2.S.CH_2.CO.NH\text{—Kemptide.}$$

The success of using this procedure to link different monoclonal antibodies to Kemptide is shown by the results of trace-labelling studies summarised in Table I. After conjugation to Kemptide, monoclonal antibodies OX7, HMFG-2 and H17E2 were able, in the presence of protein kinase, to accept approximately 2 phosphate groups per IgG molecule from ATP, whereas the unconjugated antibodies were not labelled at all. The R10-Kemptide conjugate could only be labelled with 0.7 phosphate groups per IgG molecule. The results obtained with the conjugates minus enzyme (Table I) clearly show that the labelling of conjugates is not due to an artifactual non-specific association of $^{32}$P-$\gamma$-ATP with the conjugate.

Analysis of OX7-$^{32}$P-Kemptide by HPLC showed that 95% of the radioactivity applied to the column co-migrated with OX7-Kemptide. Both the $^{32}$P-labelled and unlabelled conjugates had a mobility the same as OX7 and showed no evidence of the presence of significant amounts of dimers or larger polymers (Figure 1). The remaining activity had the mobility of (and most probably was) unreacted $^{32}$P-$\gamma$-ATP. Further HPLC analysis of a moderately labelled OX7-$^{32}$P-Kemptide sample (3 $\mu$Ci $\mu g^{-1}$ protein) stored in 2 mg ml$^{-1}$ BSA in phosphate-buffered saline at 4°C demonstrated that there was no rapid dephosphorylation of the conjugate over a 72 h period nor was there any evidence of gross radiolytic damage to antibody by the production of labelled antibody fragments.

Kinetic studies on the rapidity of phosphorylation of antibody-Kemptide conjugates have indicated that labelling is complete within 15 min (not shown).

### *Effect of conjugation on antibody-antigen recognition*

Measurements of the binding of OX7 and OX7-Kemptide to AKR-A lymphoma cells using the FACS (Figure 2) showed that there was no detectable impairment of antigen-binding capacity after the conjugation procedure. The proportions of OX7 and OX7-Kemptide that gave 50% maximal binding was ca. 100 ng $10^{-6}$ cells. The results presented in Figure 3 demonstrate the specificity of antibody-targeted $^{32}$P-phosphate. A comparison of $^{32}$P-targeting between Kemptide conjugates of OX7 and H17E2 antibodies on AKR-A cells (Figure 3) again demonstrates that only the specific OX7 antibody will allow the localisation of the radionuclide onto the target cell. Further, OX7-$^{32}$P-Kemptide did not bind detectably to Thy 1.2-expressing EL4

**Table I** Phosphorylation of conjugates

| *Substrate* | ± *Enzyme* | *P/IgG* |
|---|---|---|
| OX7 | + | 0.0 |
| OX7-Kemptide | – | 0.0 |
| OX7-Kemptide | + | 2.2 |
| H17E2 | + | 0.0 |
| H17E2-Kemptide | – | 0.0 |
| H17E2-Kemptide | + | 2.05 |
| R10 | + | 0.0 |
| R10-Kemptide | – | 0.0 |
| R10-Kemptide | + | 0.7 |
| HMFG2 | + | 0.0 |
| HMFG2-Kemptide | – | 0.0 |
| HMFG2-Kemptide | – | 1.82 |

A variety of different antibodies and conjugates were subjected to the phosphorylation procedure with trace amounts of $^{32}$P-$\gamma$-ATP in the presence or absence of the enzyme as described in **Materials and methods**. The acid-precipitable radioactivity was measured and the ratios of phosphate groups per IgG molecules calculated.

**Figure 1** HPLC analysis of OX7-Kemptide conjugate. A 50 $\mu$l sample of the conjugate in enzyme buffer (pH 7.0) was analysed on a TSK G3000 SW column as described in **Materials and methods**. The radioactivity of 1 ml fractions was counted and the uv absorption recorded. Native OX7 had a mobility identical to that of the conjugate.

**Figure 2** Comparison of the binding of OX7 and OX7-Kemptide to AKR-A lymphoma cells measured by indirect immunofluorescence. The binding of OX7 and OX7-Kemptide to AKR-A cells was measured by indirect immunofluorescence and FACS analysis as described in **Materials and methods**. Cells treated with FITC-labelled anti-mouse IgG alone showed a mean fluorescence intensity which did not exceed 20 at any concentration.

**Figure 3** Comparison of the binding of OX7-$^{32}$P-Kemptide and H17E2-$^{32}$P-Kemptide to AKR-A lymphoma cells. To $10^6$ AKR-A cells ($10^7$ ml$^{-1}$ PBS containing 2 mg ml$^{-1}$ BSA and 0.02% sodium azide) were added 100 $\mu$l of either conjugate at various concentrations. The cells were kept on ice for 1 h and then pelleted through 100 $\mu$l oil (a blend of 84% 550 and 16% 200/2 cs Silicone oil (Dow Corning)) by centrifugation for 90 sec at 12000 $g$. The centrifuge tubes were frozen on dry ice, the tips containing the cell pellets removed and counted in 2 ml Optiphase.

cells (results not shown). Similar studies with H17E2-Kemptide on placental alkaline phosphatase-expressing HEp2 cells and R10-Kemptide on glycophorin-expressing K562 cells have also shown no impairment of antibody function after conjugation (results not shown).

*Stability of conjugates in plasma and blood clearance of the OX7-Kemptide conjugates*

Incubation of a $^{32}P$-labelled bovine Ig-Kemptide conjugate in samples of murine, rat and human plasma for up to 64 h (see **Materials and methods**) gave no indication of dephosphorylation of the conjugate indicating the absence of any Kemptide-recognising phosphatases in the isolated plasma of humans, rats or mice.

In the murine blood clearance study, plasma samples were taken at various time intervals following the injection (i.v.) of either OX7-$^{32}P$-Kemptide or $^{125}I$-OX7-Kemptide into mice, and either assessed for acid-precipitable radioactive material or analysed by HPLC. The half-life of OX7-$^{32}P$-Kemptide in the blood as assessed by acid-precipitation was 2.0 days for the $\beta$-phase clearance (Figure 4). This is much shorter than the 4.0 day half-life for $^{125}I$-labelled OX7 (Figure 4). Since in parallel studies, $^{125}I$-labelled OX7-$^{31}P$-Kemptide was found to have a half-life of 2.8 days, it seems likely that the shorter half-life of OX7-$^{32}P$-Kemptide is related to the enhanced clearance of the conjugate compared with the native antibody rather than being the result of dephosphorylation. HPLC analysis of the plasma samples showed that the $^{32}P$ remained associated with protein which co-migrated with OX7-antibody (not shown).

**Figure 4** Blood clearance rates of $^{125}I$-OX7, OX7-$^{32}P$-Kemptide and $^{125}I$-OX7-Kemptide. The clearance of OX7 and OX7-Kemptide from the murine bloodstream was measured as described in **Materials and methods**. The radioactivity in the plasma is expressed as a percentage of the $T_0$ value, the level at 10 min post injection.

### Discussion

Kemptide, a synthetic heptapeptide substrate for protein kinases, has been covalently bonded to monoclonal antibodies to provide phosphorylatable immunoglobulins which can be used to assess the tumoricidal potential of antibody-targeted $^{32}P$. The conjugation method uses the protein-protein coupling chemistry developed by Rector *et al.* (1978) which has been employed recently to produce antibody-ricin immunotoxins (Thorpe *et al.*, 1984). Four different monoclonal antibodies as well as a bovine immunoglobulin fraction have been conjugated to Kemptide by this process and as a result are able to accept 1–2 phosphate groups per immunoglobulin molecule from ATP in the presence of appropriate kinases. Using carrier-free $^{32}P$-$\gamma$-ATP (specific activity $>5000\,Ci\,mmol^{-1}$), phosphorylated antibodies with a specific activity of $10\,\mu Ci\,\mu g^{-1}$ have been achieved. Potentially this could be increased to a theoretical maximum of $30$–$60\,\mu Ci\,\mu g^{-1}$ if saturation of all the available phosphorylation sites were attained.

None of the antibody-Kemptide conjugates synthesised so far have shown any reduction in antigen-binding activity and thus it seems likely that this conjugation procedure could be usefully extended to other monoclonal antibodies and to non-immunoglobulin proteins as well. The ability of antibody-Kemptide conjugates to act as substrates for protein kinase would also suggest that there is no major impairment of Kemptide phosphate acceptor function although this has not been assessed in detail. The possibility that the phosphorylation of conjugates was a consequence of the conjugation procedure revealing hitherto occluded phosphorylation sites in the antibody molecule was discounted since antibodies that had been through the conjugation procedure in the absence of Kemptide could not be phosphorylated.

Studies on the antibody-directed localisation of $^{32}P$ onto cells clearly showed that the binding of $^{32}P$-labelled antibody required the presence of the antigen recognised by the targeting antibody. Non-specific targeting of $^{32}P$ by antibody-Kemptide conjugates was found to be negligible.

Studies of the stability of antibody-$^{32}P$-Kemptide conjugates in plasma *in vitro* show that there is no rapid dephosphorylation of the conjugate by putative plasma phosphatases. However investigation of the blood clearance of OX7-$^{32}P$-Kemptide in mice showed that the $\beta$-phase half-life of the conjugate was only 2.0 days as compared with 4.0 days for $^{125}I$-labelled OX7. Parallel studies with $^{125}I$-labelled OX7-$^{31}P$-Kemptide showed that its blood half-life was only 2.8 days. This demonstrates that OX7-Kemptide is cleared more rapidly than native antibody and that the enhanced clearance of the conjugate is not the result of dephosphorylation. The 0.8 day discrepancy between $^{32}P$ and $^{125}I$-measured half-lives can be accounted for by the presence of unconjugated OX7 in the conjugate preparation: the longer blood survival of $^{125}I$-labelled unconjugated OX7 would contribute to the half-lives measured by $^{125}I$ but would not, of course, have affected those based on the measurement of $^{32}P$. The enhanced clearances of antibody-Kemptide conjugates would suggest that even apparently minor modifications to the antibody structure can lead to a faster removal of immunoglobulin from the blood. Since HPLC analysis showed an absence of significant amounts of dimers or higher polymers, the enhanced clearance cannot simply be due to the removal of such aggregates.

Although theroretically it would be more attractive to use a linkage involving the more stable carbon phosphorus bond, the preparation of the appropriate reagents at high specific activities presents special difficulties which will not be easily resolved. The novel procedure we describe here has the virtue that it makes use of readily available derivatives of $^{32}P$ and should allow the therapeutic potential of the targeted radioisotopes to be assessed. Initial studies (to be published elsewhere) have shown that these conjugates can direct $^{32}P$ to subcutaneous AKR-A tumours achieving concentrations of up to 12.4% of the injected dose/gram. Thus we believe that antibody $^{32}P$-Kemptide conjugates represent a new avenue in the development of antibody-targeted tumoricidal agents.

The authors wish to thank Audrey Becket for typing the manuscript.

### References

ANONYMOUS. (1967). Today's drugs: Radioactive phosphorus. *Br. Med. J.*, **ii**, 225.

BEAVO, J.A., BECHTEL, P.J. & KREBS, E.G. (1974). Preparation of homogeneous cyclic AMP-dependent protein kinase(s) and its subunits from rabbit skeletal muscle. *Methods in Enzymology*, **38**, 299.

BOYE, E., LINDEGAARD, M.W., PAUS, E., SKRETTING, A., DAVY, M. & JAKOBSEN, E. (1984). Whole-body distribution of radioactivity after intraperitoneal administration of $^{32}P$ colloids. *Br. J. Radiol.*, **57**, 395.

CARLSSON, J., DREVIN, H. & AXEN, R. (1978). Protein thiolation and reversible protein-protein conjugation. *Biochem. J.*, **173**, 723.

COURTENAY-LUCK, N., EPENETOS, A.A., HALNAN, K.E. & 17 others (1984). Antibody-guided irradiation of malignant lesions: Three cases illustrating a new method of treatment. *Lancet*, **ii**, 1441.

DE LA HOUSSAYE, B.A. & MASARACCHIA, R.A. (1983). Standardization of the assay for the catalytic subunit of cyclic AMP-dependent protein kinase using a synthetic peptide substrate. *Anal. Biochem.*, **128**, 54.

EPENETOS, A.A., COURTENAY-LUCK, N., PICKERING, D. & 4 others (1985). Antibody-guided irradiation of brain glioma by arterial infusion of radioactive monoclonal antibody against epidermal growth factor receptor and blood group A antigen. *Br. Med. J.*, **290**, 1463.

ETTINGER, D.S., ORDER, S.E., WHARAM, M.D., PARKER, M.K., KLEIN, J.L. & LEICHNER, P.K. (1982). Phase I–II study of isotopic immunoglobulin therapy for primary liver cancer. *Cancer Treat. Rep.*, **66**, 289.

HALPERN, S.E., STERN, P.L., HAGAN, P.H. & 7 others (1981). Radiolabelling of monoclonal antitumour antibodies: Comparison of I-125 and In-111-anti-CEA with Ga-67 in a nude mouse-human colon tumour model. *Clin. Nucl. Med.*, **6**, 453.

HNATOWICH, D.J., CHILDS, R.L., LANTEIGNE, D. & NAJAFI, A. (1983). The preparation of DTPA-coupled antibodies radiolabeled with metallic radionuclides: An improved method. *J. Immunol. Meth.*, **65**, 147.

HNATOWICH, D.J., LAYNE, W.W., CHILDS, R.L. & 4 others (1983). Radioactive labeling of antibody: A simple and efficient method. *Science*, **220**, 613.

HNATOWICH, D.J., VIRZI, F. & DOHERT, P.W. (1985). DTPA-coupled antibodies labeled with Yttrium-90. *J. Nucl. Med.*, **26**, 503.

KEMP, B.E., GRAVES, D.J. & BENJAMINI, E. (1976). Synthetic peptide substrates of the cAMP-dependent protein kinase. *Fed. Proc.*, **35**, 1384.

LARSON, S.M., CARRASQUILLO, J.A., KROHN, K.A. & 8 others (1983). Localization of $^{131}$I-labelled p97-specific Fab fragments in human melanoma as a basis for radiotherapy. *J. Clin. Invest.*, **72**, 2101.

LANTEIGNE, D. & HNATOWICH, D.J. (1984). The labeling of DTPA-coupled proteins $^{99m}$Tc. *Int. J. Appl. Radiat. Isot.*, **35**, 617.

ORDER, S.E., KLEIN, J.L. & LEICHNER, P.K. (1981). Antiferritin IgG antibody for isotopic cancer therapy. *Oncology*, **38**, 154.

ORDER, S.E., KLEIN, J.L., LEICHNER, P.K., FRINCKLE, J., LOLLO, C. & CARLO, D.J. (1986). $^{90}$Yttrium antiferritin – a new therapeutic radiolabeled antibody. *Int. J. Radiat. Oncol. Biol. Phys.*, **12**, 277.

PAXTON, R.J., JAKOWATZ, J.G., BEATTY, J.D. & 5 others (1985). High-specific-activity $^{111}$In-labeled anticarcinoembryonic antigen monoclonal antibody: Improved method for the synthesis of diethylenetriaminepentaacetic acid conjugates. *Cancer Res.*, **45**, 5694.

RECTOR, E.S., SCHWENK, R.J., TSE, K.S. & SEHON, A.H. (1978). A method for the preparation of protein-protein conjugates of predetermined composition. *J. Immunol. Meth.*, **24**, 321.

SCHEINBERG, D.A., STRAND, M. & GANSOW, O.A. (1982). Tumour imaging with radioactive metal chelates conjugated to monoclonal antibodies. *Science*, **215**, 1511.

SULLIVAN, D.C., SILVA, J.S., COX, C.E. & 4 others (1982). Localization of $^{131}$I-labelled goat and primate anticarcinoembryonic antigen (CEA) antibodies in patients with cancer. *Invest. Radiol.*, **17**, 350.

THORPE, P.E., ROSS, W.C.J., BROWN, A.N.F. & 4 others (1984). Blockade of the galactose-binding sites of ricin by its linkage to antibody: Specific cytotoxic effects of the conjugates. *Eur. J. Biochem.*, **140**, 63.